# Editorial: The role of early-life nutrition and metabolism in brain development and adult behavior

**DOI:** 10.3389/fnins.2023.1308183

**Published:** 2023-12-18

**Authors:** Gonzalo H. Olivares, Patricio Olguín

**Affiliations:** ^1^Escuela de Kinesiología, Facultad de Medicina y Ciencias de la Salud, Center for Integrative Biology (CIB), Universidad Mayor, Santiago, Chile; ^2^Departamento de Neurociencia, Biomedical Neuroscience Institute (BNI), Facultad de Medicina, Universidad de Chile, Santiago, Chile; ^3^Programa de Genética Humana, Instituto de Ciencias Biomédicas (ICBM), Facultad de Medicina, Universidad de Chile, Santiago, Chile

**Keywords:** early-life nutrition, brain development, adult behavior, synaptic plasticity, brain morphology

Early-life nutritional environment impacts neural development and can have long-lasting consequences on adult complex behaviors, such as sleep, locomotion, memory, and learning (Bale et al., 2010; Crossland et al., 2017; Olivares et al., 2023). In humans, prenatal severe nutritional restriction affects intellectual functioning (Li et al., 2016) and increases the risk of suffering from affective disorders and mental illness (Brown et al., 2000; Hulshoff Pol et al., 2000). The origin of these behavioral disorders might be associated with effects on early brain development and epigenetic changes that affect the function of the adult nervous system (Zúñiga-Hernández et al., 2023). Nevertheless, early-life nutrition's molecular, cellular, and developmental mechanisms must be better understood. In this Research Topic, two articles and one review address this question.

Thau-Zuchman et al. present their study on how phenylalanine accumulation in individuals suffering from phenylketonuria leads to white matter damage. *Phenylketonuria* is an autosomal recessive genetic disease that results from mutations in the phenylalanine hydroxylase coding gene. If not treated early in newborns, phenylalanine accumulation leads to severe intellectual disability and psychiatric and movement disorders (Hillert et al., 2020; van Spronsen et al., 2021). The authors use a series of increasing complexity *in vitro* experiments, from primary cell cultures to organotypic slide cultures, to demonstrate that phenylalanine exposure in cell cultures did not affect viability but in high concentration resulted in oligodendrocyte differentiation. Importantly, their results show that microglial activation may precede demyelination in cerebellar organotypic slices exposed to high concentrations of phenylalanine, supporting the idea that microglial activation may play a critical role in the demyelination of white matter.

Dun et al. contribute with more evidence to their previous observation that the developing serotonin system is sensitive to a Western-style diet (DeCapo et al., 2019). Using a series of immunohistochemical studies, they show that a Western-style diet strongly affects the density of serotonin-producing neurons, which may decrease global serotonin abundance in the brain and strongly impact offspring behavior. Finally, Fung summarizes and discusses the neurological consequences of intrauterine growth restriction (IUGR) caused by uteroplacental insufficiency in humans, its impact on the development of the dentate gyrus of the hippocampus, using an IUGR mouse model developed by her group, and the critical period of synaptic plasticity during postnatal neuronal development and its role in the excitatory-inhibitory balance in the developing brain.

In addition to mechanistic studies, two articles focus on developing tools and techniques that better predict the effects of early-life malnutrition on short-term and long-term cognitive performance. First, Li et al. present a model to predict short-term neurodevelopmental impairment in preterm infants. Using multivariate logistic regression, the authors demonstrate that extrauterine growth restriction, among other factors such as gestational age, vaginal delivery, and hyperbilirubinemia, predicts neurodevelopment impairment. Furthermore, Razzaq et al. explored semiquantitative and spectral quantitative EEG to assess the lifelong effects of protein-energy malnutrition in the Barbados Nutrition Study. This 50-year-old longitudinal study has followed individuals who suffered from protein-energy malnutrition only during their 1st year of life (Galler et al., 1983). They show that the complementary use of these techniques predicts the cognitive decline of individuals exposed to a low-protein diet during development more accurately.

Overall, this Research Topic covers two main research areas on early-life nutrition, neural development, and behavior: mechanistic and prediction models. Essential efforts to unveil molecular and cellular mechanisms and to develop more accurate prediction models will be critical in designing potential therapeutic strategies to modify abnormal neurodevelopmental trajectories leading to altered behaviors and brain diseases.

## Author contributions

GO: Writing – review & editing. PO: Writing – original draft, Writing – review & editing.
